# Moral Foundations and Patient‐Centered Care in a Brazilian Maternity Ward: A Survey Study

**DOI:** 10.1111/jep.70384

**Published:** 2026-03-01

**Authors:** Isabella de Melo Rodrigues Franco, Aline Albuquerque, Cristina Ortiz Sobrinho Valete

**Affiliations:** ^1^ Department of Medicine São Carlos Federal University São Carlos Brazil; ^2^ Post‐graduate Program of Bioethics University of Brasilia Brasilia Brazil

**Keywords:** healthcare, medical ethics, patient‐centered care

## Abstract

**Aim:**

To investigate the relation between moral foundations and patient‐centered care in health professionals who work in the delivery room and in the rooming‐in.

**Design:**

Single‐center quantitative survey study.

**Methods:**

This survey was conducted in a Brazilian maternity ward. The Moral Foundations Questionnaire (MFQ‐20) and the Care in Dialogue Competence Scale (CDCS) were administered to health professionals working in the delivery room and rooming‐in areas. Analysis included descriptive statistics and Spearman correlations. Data were analyzed using Stata version 19.0.

**Results:**

A total of 80 health professionals were included, and the median age was 34 years (IQR: 28.5–44). Cronbach's alpha of the MFQ‐20 was 0.8480, and CDCS was 0.8499. In the MFQ‐20, fairness and care were the domains with the highest median, and in the CDCS, communication and dialogue with the patient. MFQ‐20 and CDCS were correlated (0.54; *p* < 0.001). Purity presented the highest domain‐specific correlation with CDCS (0.48; *p* < 0.0001).

**Conclusion:**

In this maternity ward, health professionals' moral foundations are correlated with patient‐centered care. Although fairness and care were the MFQ‐20 domains with the highest medians, purity was the domain most strongly correlated to CDCS.

**Impact:**

Clinical practice requires health professionals to use moral dimensions that are part of their individual essence, and this can correlate with patient‐centered care, a dimension of quality care.

****Patient or** Public C**ontribution**:**

These results contribute to a better understanding of the relations between moral foundations and patient‐centered care in neonatology. By highlighting moral motivations strongly associated with patient‐centered care, we can strengthen it.

## 1 Introduction

In the last decades, there has been an improvement in neonatal care due to technological advances, resulting in a worldwide reduction in neonatal mortality [1]. On the other hand, the resulting care continues to present a disconnection between health professionals and patients, characterized by a detachment between them [2]. This detachment has been criticized by theorists from the clinical empathy field, based on studies about the benefits of emotional and cognitive empathy in healthcare. In this connection, new bioethical approaches have been establishing the importance of developing ethical competences among health professionals, notably to help them improve their skills in identifying the moral aspects of their behavior and judgments, as well as moral disagreements. The morality of health professionals' behavior and decisions has a significant impact on patient‐centered care. Therefore, it is crucial to study their interactions, as proposed by Healthcare Bioethics, a new theoretical‐normative framework for the clinical context [3].

This framework underscores the patient‐health professional relationship and its essential elements, such as clinical empathy and patient‐centered care. Therefore, Healthcare Bioethics seeks to foster the exchange between them [4]. Patient‐centered care is a pillar of quality care and is defined by the Institute of Medicine as care that respects and responds to patients' preferences, needs, and values, ensuring that patients' values drive all clinical decisions [5]. It is a challenge in neonatal care, especially considering the neonate's characteristics and vulnerability. Notably, the moral judgments of health professionals may correlate with this model of care.

Neonatal care requires a health professional with both scientific knowledge and clinical empathy to provide a comfortable and high‐quality encounter, addressing patients' needs effectively, without disregarding what is considered morally correct [6]. Morality can be defined as the various conceptions people have of right or wrong, and is both individual and socially constructed. According to Haidt, it is partly innate and partly learned from others and society at large, and so, it can be socially modified [7, 8]. Considering the healthcare scenario, and that morality may be socially influenced, shaping the idea of right and wrong, health professionals' practices and behaviors are influenced by moral aspects. In fact, practices and morality are imbricated, although some meanings are difficult to understand. But clinical practices require attending to the appropriate rules, and practices are where the moral action is [9].

The Moral Foundations Theory (MFT) states that moral judgements are innate intuitions supported by five foundations: harm/care, fairness/reciprocity, ingroup/loyalty, authority/respect, and purity/sanctity. MFT is the most frequently studied moral theory, and it was associated with health professionals' personality and patients' satisfaction. [10, 11] Haidt argues that moral intuitions drive our later justifications: first, a moral stance is chosen intuitively, and we later rationalize it, if necessary [7]. The use of questionnaires has become a practice in research to map out people's morality [12].

There is a lack of studies on patient‐centered care and moral foundations in neonatal care in Brazil. Therefore, this study aimed to investigate the relation between moral foundations and patient‐centered care in health professionals who work in the delivery room and in the rooming‐in.

## 2 Methods

### 2.1 Study Design and Setting

A single‐center survey study design was used in this study, conducted in a maternity ward and delivery room at a public tertiary maternity hospital located in São Paulo, Brazil. The maternity ward makes around 250 deliveries per month.

### 2.2 Study Participants and Sampling

All categories of health professionals who worked in the rooming‐in or the delivery room and agreed to participate were included through convenience sampling. Exclusion criteria were absence from work during data collection. The multidisciplinary team has 90 health professionals. Sample size was estimated at Raosoft (https://www.raosoft.com/samplesize.html) considering a power of 80%, a 50% response distribution, a maximum estimation error of 5%, and a confidence level of 95%, resulting in a minimum of 74 participants.

### 2.3 Data Collection

Ethical approval was obtained from the Research Ethics Committee of the institution before data collection, and all participants signed an informed consent form. Data collection occurred from February to May 2025. An explanation about the study, its purposes, and the questionnaire was provided for the eligible health professionals. The questionnaire was built in REDCap (Research Electronic Data Capture), self‐administered during work shift, and included information about health professionals' characteristics (age, gender, specialty, and years of clinical experience), the two‐part Moral Foundations Questionnaire (MFQ‐20), and the Care in Dialogue Competence Scale (CDCS) [13]. Questionnaire completion took about 10–15 min. The questionnaire was checked for completeness, and confidentiality was guaranteed through REDCap.

### 2.4 Measures

**Figure 1:**
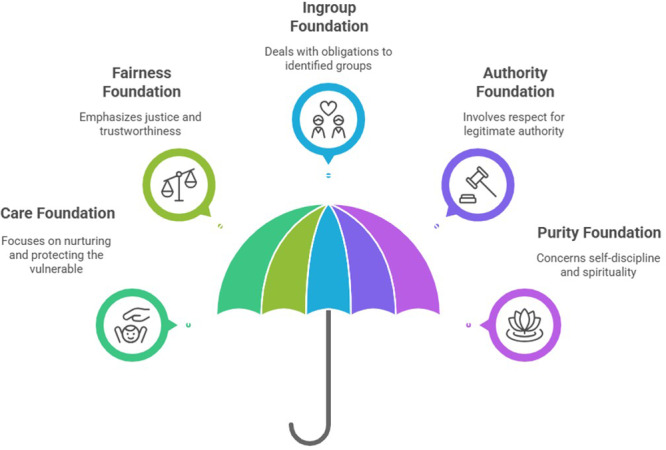
The MFQ‐20 five foundations' concerns (Figure made with napkin. ai).

Moral foundations were evaluated using the MFQ‐20, a free questionnaire available in Portuguese (https://moralfoundations.org/questionnaires/) with 20 items on a Likert scale. The first part refers to the importance of the questions “When you decide whether something is right or wrong, to what extent are the following considerations relevant to your thinking?”, ranging from 0‐not at all relevant to 5‐extremely relevant. This part measures the relevance of the items. The second part refers to the agreement with the statements, ranging from 0‐*strongly disagree* to 5‐*strongly agree*, measuring judgment. Figure 1 shows the five foundations (domains), named care, fairness, ingroup, authority, and purity [9].

Patient‐centered care was evaluated by the CDCS. The CDCS is free for use, is translated to Brazilian Portuguese, adapted, and validated in Brazil [14], and has 11 items in a Likert scale ranging from 1‐*never* to 5‐*always*. It is divided into three domains named communication and dialogue with the client, shared care management with the client, and proactive behavior. The CDCS refers to patients as clients. For this study, we will consider the term patient.

In this study, Cronbach's alpha of the MFQ‐20 was 0.8480, and of the CDCS was 0.8499.

### 2.5 Data Analysis

Stata program version 19.0 (Stata Corp, L.C.) was used to analyze data. Normality was calculated by the Shapiro–Wilk test. Numeric variables are presented as median and interquartile range (IQR), and proportions as percentages. The primary outcome was patient‐centered care. Differences between medians were analyzed using the Mann‐Whitney test, and proportions were analyzed using the chi‐squared test. The Kruskal‐Wallis test was used to compare more than two medians, with the Dunn post‐hoc test. To investigate the association between the MFQ‐20 and CDCS, a Spearman correlation analysis was performed. We observed the MFQ‐20 domains that correlated with global CDCS and with the three domains, considering *a* > 0.30 cut‐off (medium to large correlations) [15]. The highest correlations were identified. The results are presented in tables and figures. This study follows the CROSS checklist for survey studies [16]. A *p*‐value of 0.05 was considered.

## 3 Results

A total of 80 health professionals were included (88.8% of all the team); 79 (98.7%) were female, and the median age was 34 years (IQR 28.5‐44). Seventeen (21.2%) were nurses, 39 (48.7%) were nurse assistants, 23 (28.7%) were physicians, and one (1.4%) was a physiotherapist. Twenty‐nine (36.2%) worked in the neonatal area for < 1 year, 15 (18.8%) between 1 and 2 years, 22 (27.5%) between 3 and 10 years, and 14 (17.5%) for more than 10 years.

### 3.1 The MFQ‐20

**Figure 2:**
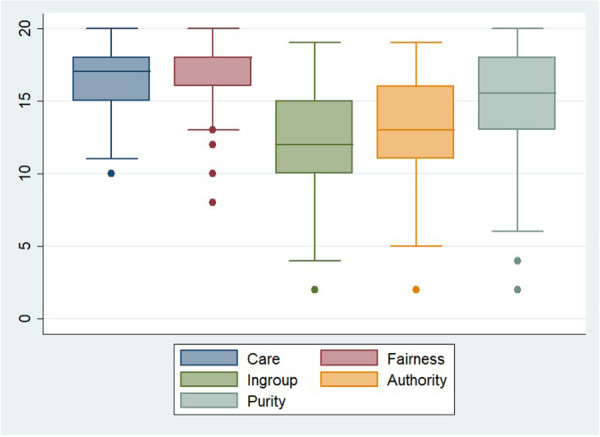
Medians of the MFQ‐20 domains.

Median MFQ‐20 was 75 (IQR: 66.0–83.5). The median MFQ‐20 scores for nurses and physicians were 73 (IQR: 62–79) and 71 (IQR: 64–79), respectively, with no significant difference (*p* = 0.84). There was no difference considering the years of experience (*p* = 0.42). Figure 2 shows that the domains presenting higher values were fairness, with a median of 18 (IQR: 16–18), and care, with a median of 17 (IQR: 15–18).

The Kruskal‐Wallis test revealed that the groups were different (*p* = 0.0001). Dunn's test revealed there was no difference between the domain care and fairness (*p* = 0.10), ingroup and authority (*p* = 0.05).

### 3.2 The CDCS

**Figure 3:**
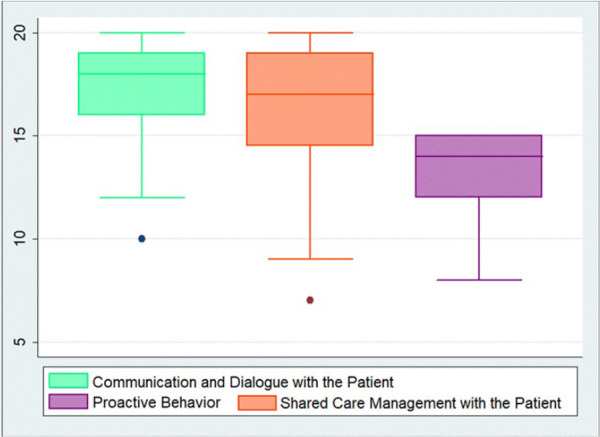
Medians of the CDCS domains.

Median CDCS was 48 (IQR: 43.5–51.0). The median CDCS for nurses was 45 (IQR: 38–52) and for physicians 48 (IQR: 43–50) without differences (*p* = 0.90). There was no difference considering the years of experience (*p* = 0.37). Figure 3 shows that the domain communication and dialogue with the patient had a higher median, compared to the others (*p* = 0.002).

### 3.3 The MFQ‐20 and CDCS Correlations

MFQ‐20 and CDCS were correlated (0.54; *p* < 0.001). Table 1 shows that the highest correlation was between CDCS and the domain purity. Ingroup was the domain with the highest correlation with communication and dialogue with the patient, purity with shared care management with the patient, and purity and care with proactive behavior.

**Table 1:** Spearman's correlations between MFQ‐20 domains and CDCS variables (*n* = 80).

MFQ‐20 Domain	Global CDCS	Communication and dialogue with the patient	Shared care management with the patient	Proactive behavior
Care	0.42 (0.25–0.60; 0.0001)*	0.32 (0.13–0.51; 0.003)	0.31 (0.12–0.49; 0.0052)	0.42 (0.23–0.62; 0.0001)
Ingroup	0.43 (0.24–0.61; 0.0001)	0.42 (0.24–0.61; 0.0001)	0.30 (0.10–0.50; 0.0060)	0.37 (0.17–0.56; 0.0008)
Purity	0.48 (0.34–0.63; < 0.0001)	0.38 (0.19–0.57; 0.0005)	0.40 (0.22–0.58; 0.0002)	0.42 (0.24–0.59; 0.0001)
Authority	0.37 (0.18–0.57; 0.0006)	0.38 (0.20–0.56; 0.0004)	0.22 (0.02–0.42; 0.0446)	0.34 (0.14–0.54; 0.0017)
Fairness	0.41 (0.23–0.59; 0.0002)	0.37 (0.16–0.58; 0.0007)	0.31 (0.12–0.50; 0.0046)	0.34 (0.15–0.53; 0.0018)
Cronbach alpha	0.8499	0.8013	0.7770	0.6876

* 95% Confidence Intervals and *p*‐values.

## 4 Discussion

This study was conducted to investigate the relation between moral foundations and patient‐centered care in health professionals who work in the delivery room and in the rooming‐in. The study revealed that MFQ‐20 and CDCS are correlated. It was noted that the domains of the MFQ‐20 that most correlated with the CDCS domains were in order: purity, ingroup, and care.

The Cronbach alpha for both the MFQ‐20 and the CDCS reflects good reliability for both questionnaires [17]. The MFQ‐20 was recently applied to nurses in neonatal intensive care and presented a Cronbach alpha < 0.70 [18]. The value of the Cronbach alpha observed in the present study is interesting. Neither questionnaire has been frequently applied to health professionals who work with neonates, and the results of this study suggest that both are reliable for this scenario, and other studies could apply the MFQ‐20 and CDCS to neonatal care.

The domains of the MFQ‐20 revealed higher values for fairness, without a statistical difference with care. From the fairness domain comes a universally recognized virtue—justice. The care domain concerns caring, nurturing, kindness, compassion, and protecting the vulnerable [19]. In a study that investigated the MFQ‐20 in health professionals, it was observed that higher mean values were obtained for the domain care, followed by fairness [20]. Graham et al. observed higher punctuations for the domain care, followed by fairness, in a different scenario [10]. Accordingly, Barr observed in the intensive care unit that the MFQ‐20 domains care and fairness presented higher and equal medians [18]. The fact that health professionals value justice and care is not surprising, considering the neonate's vulnerability, and may represent that health professionals tend to “protect” neonates. This is in agreement with Haidt's statement that harm/care evolved from a maternal sensitivity to suffering in offspring. Also, the first two foundations—care and fairness—are interrelated and focus on respect for the rights of others, based on an individualized approach. When people consider the harm they can cause or the fairness of their actions, they are motivated to act more selflessly, and so are health professionals [19]. This seems to point out that justice and care are important moral motivations in the team studied.

In the CDCS, the domain of communication and dialogue with the patient had the highest punctuation. This domain concerns implementing and discussing the care plan with the patient, and this result may represent the strength of interpersonal aspects of caring communication, which may also reflect particular aspects of care. In neonatology, the care plan is discussed with the family, but it should take into account the neonate's needs, which can be recognized by proper nonverbal communication. Differently, Silva et al. analyzed health professionals' perspectives by applying the CDCS and observed higher mean scores for variables from the proactive behavior domain, followed by communication and dialogue with the patient [21]. The results observed in the present study suggest that communication with the patient is a strength in patient‐centered care among health professionals working with neonates.

All MFQ‐20 domains and CDCS were correlated. The highest correlation was between CDCS and domain purity. This may represent that self‐discipline, personal growth, modesty, and spirituality may help support patient‐centered care. Also, this result agrees with Duggan et al. who described the moral commitments that underlie patient‐centered care, recognizing its moral value, although they did not apply the MFQ‐20 [22]. Barr observed that purity was correlated with untruthful care (‘ignore situations in which parents have not been given adequate information to ensure informed consent’), shame (‘feeling humiliated, ridiculous, embarrassed, helpless, paralysed, and self‐consciouness’) and guilt (‘have guilt, regret, or worry about hurting or injuring someone’), in a study that compared the MFQ‐20 with moral distress and self‐conscious moral emotions [18]. This discussion is important as purity is the most contested of Haidt's foundations, and difficult to understand. The Moral Foundations Theory assumes that purity is a universal, coherent psychological concept and a separate cognitive moral mechanism. But a dyadic morality has been argued by other theories, and we should acknowledge this, especially related to moral psychology, that purity is an umbrella term that can mean different and heterogeneous ideas, and the term is grounded in culturally constructed perceptions of harm, which suggests that there are interrelations between the domains. Indeed, perceptions of harm and judgments of wrongness go hand‐in‐hand [23]. Gray et al. stated that it is easier to understand what purity isn't: obvious interpersonal harm [23]. It is not our purpose to close this debate, but our results suggest that in the context of CDCS and for this sample of health professionals, the morality of relationships is evaluated through a filter of purity and not merely of utility or absence of harm. We understand this result is in line with Graham et al. who considered foundations statistically distinct. They stated the MFQ‐20 as an open, in motion, and revisable theory [10]. In the neonatal scenario, emphasis on purity may lead individuals to see a neonate's vulnerability and fragility as a state of sacredness and innocence, and this correlates with patient‐centered care.

Considering the domains' correlations between the MFQ‐20 and the CDCS, the ingroup presented the highest correlation with Communication and dialogue with the patient. This domain refers to obligations to groups with which one identifies. Virtues and emotions concerning trust, patriotism, heroism, and sacrifice arise in this foundation [19]. It seems that health professionals' sense of group influences their communication with the patient. Barr also observed that the ingroup was correlated with compromised care [18]. Lisman & Holman applied the empathy scale developed by Ferreira Duarte et al. [24], composed of three dimensions: perspective taking, compassionate care, and a cognitive dimension (e.g., ‘Understanding body language is as important as verbal communication in physician‐patient relationships’). They observed that the cognitive dimension of empathy correlated with the domain ingroup [20]. Although it was not our purpose to explore empathy and moral foundations, it is recognized that empathy is a cornerstone of patient‐centered care. Reinforcing these results, Dlamini et al. observed that among nurses, communication skills and teamwork were predictors of patient‐centered care [25]. In the present study, the observed result suggests that in the neonatal scenario studied, health professionals' group obligations influence their communication with patients.

Findings from this study revealed that purity had the highest correlation with shared care management with the patient. The purity domain concerns the widespread idea that the body is a temple that can be desecrated by immoral activities and contaminants. Purity has not only been described with reference to our physical world (e.g., pathogens or sex) but also our metaphysical (or spiritual) world. Descriptions of purity as spiritual integrity (vs*.* defilement) can be traced back to early descriptions of the construct [19]. This may reflect that those who strongly endorse the spiritual dimension of care also support patient initiatives as a way of promoting other healthcare dimensions.

Care and purity presented the highest correlation with proactive behavior, revealing a more collaborative behavior to attend to patients' demands as purity and care increase. Given the smaller number of items in this domain compared with other CDCS domains, the slightly lower internal consistency of proactive behaviour (Cronbach's *α* = 0.687) may have influenced these associations, which should therefore be interpreted with caution. Regarding the meaning of proactive behavior, there are many frameworks suggesting that it is composed of anticipation, planning, and action. Dayeong et al. conducted a concept analysis and concluded that proactive nursing attributes included surveilling to surface problems, employing practices that go beyond the established protocols, adjusting a path toward the care goal, and arranging resources in advance [26]. Coelho et al. observed in a scoping review that investigated nurses' ethical practices that care ethics includes virtues such as humility, trust, prudence, empathy, diligence, and others, reinforcing the relation between proactiveness and moral aspects [27]. It was observed in a systematic review that job crafting is the most consistently studied proactive behavior in healthcare settings. Other behaviors were active problem solving, voice, extra‐role behaviors, and idiosyncratic deals. Job crafting refers to the way health professionals reshape their own tasks, relationships, and the way they view work to make it more meaningful and aligned with their interests, skills, and values, and has implications for work engagement, performance, and patients' care plans [28]. Considering that care concerns kindness, compassion, and purity concerns self‐discipline, personal growth, modesty, and spirituality, we suggest that these behaviors promote proactiveness among health professionals in the studied center.

## 5 Limitations of the Study

This study has limitations. First, the applied scales were not created specifically for the neonatal scenario. Second, as a single‐center study, its results should not be extrapolated to all maternity wards. Third, almost all health professionals are female, and this gender homogeneity may have interfered with results, considering gender differences in moral foundations research. Fourth, the proactive behaviour domain showed slightly lower internal consistency, and findings involving this domain should therefore be interpreted with caution. Finally, the measurement of self‐perception may not predict people's behavior, and the correlations do not imply causality. On the other hand, as a pioneering study, the scales revealed good consistency, suggesting that moral foundations correlate with patient‐centered care in neonatology and inspiring future studies to explore this issue, aiming to strengthen patient‐centered care in the neonatal scenario.

## 6 Conclusions

In this study, it was observed that health professionals' moral foundations are correlated with patient‐centered care. Purity was the domain most correlated to CDCS. Stronger correlations were observed between ingroup and communication and dialogue with the patient, purity and shared care management with the patient, and purity and care with proactive behavior. This suggests that care, the spiritual dimension of care, and the group obligations of health professionals are important values influencing patient‐centered neonatal care. Knowing the moral foundations that these health professionals value and those strongly associated with patient‐centered care can help understand their moral motivations and identify how to improve clinical practice.

## Conflicts of Interest

The authors declare no conflicts of interest.

## Data Availability

The data that support the findings of this study are available on request from the corresponding author. The data are not publicly available due to privacy or ethical restrictions.
